# Treatment of Bursa Patellæ Amplificata

**Published:** 1880-02

**Authors:** 


Treatment of Bursa Patellæ Amplificata.—Thinking it might be of use or benefit to my medical brethren, I am induced to report a case of housemaid's knee and the treatment I adopted. Miss A. C., aged about forty, a most exemplary housekeeper, was in the practice of sweeping into a dust-pan little particles of dust, etc., that might happen to be on the floor of her rooms, and while thus engaged would rest her weight upon her left knee, the result of which was the production of the disease in description. Quite a large tumor had formed when I was called to attend the case; a creaking sound was produced by rubbing or pricking the tumor. Having in various cases seen the great benefit derived from absorption produced by pressure, I concluded to give it a trial in this case. Painting the surface of the tumor with tincture of iodine, it having been absorbed, I applied pressure by drawing tightly over the tumor strips of strong adhesive plaster.

This treatment was conducted for two weeks with apparent benefit, the tumor having decreased considerably; now the effectiveness of this treatment seemed to be at an end, the tumor commencing again to enlarge. Then I made a round block of wood, a little larger than the tumor; the external side convex, the internal concave, slightly, and covered with sheepskin. This I applied to the tumor, upon which it was pressed firmly, by means of adhesive strips; during the night I supplemented the pressure by applying a tourniquet. Under this treatment the tumor rapidly declined, and in three weeks it had entirely disappeared. Nine months have elapsed since the cure, and as yet no traces of the tumor are visible.

Just after the cure had been effected, I was in the office of an eminent surgeon in Philadelphia, and commenced to tell him of my case of housemaid's knee; he anticipated that I desired his opinion in regard to treatment, and kindly suggested—"Cut it out." I told him there was nothing to cut out; it having been cured by absorption, produced by pressure, and described to him the means I had employed.

He replied to my description that it would return.

Well, if it returns it must be after more than nine months since it has disappeared, as that time has now elapsed. I hope any who read this article, if they have an opportunity, will give my treatment a trial.—*Medical Summary.*

